# Enzyme-Crosslinked Electrospun Fibrous Gelatin Hydrogel for Potential Soft Tissue Engineering

**DOI:** 10.3390/polym12091977

**Published:** 2020-08-31

**Authors:** Kexin Nie, Shanshan Han, Jianmin Yang, Qingqing Sun, Xiaofeng Wang, Xiaomeng Li, Qian Li

**Affiliations:** 1 School of Mechanics and Safety Engineering, Zhengzhou University, Zhengzhou 450001, China; nnnkexin@163.com (K.N.); shanshanhan775@163.com (S.H.); xiaofengwang@zzu.edu.cn (X.W.); qianli@zzu.edu.cn (Q.L.); 2 National Center for International Joint Research of Micro-nano Moulding Technology, Zhengzhou University, Zhengzhou 450001, China; 3 College of Biological Science and Engineering, Fuzhou University, Fuzhou 350108, China; jmyang@fzu.edu.cn; 4 Center for Functional Sensor and Actuator, National Institute for Materials Science, 1-1 Namiki, Tsukuba, Ibaraki 305-0044, Japan; SUN.Qingqing@nims.go.jp

**Keywords:** fibrous hydrogel, enzymatic crosslinking, soft tissue engineering

## Abstract

Soft tissue engineering has been seeking ways to mimic the natural extracellular microenvironment that allows cells to migrate and proliferate to regenerate new tissue. Therefore, the reconstruction of soft tissue requires a scaffold possessing the extracellular matrix (ECM)-mimicking fibrous structure and elastic property, which affect the cell functions and tissue regeneration. Herein, an effective method for fabricating nanofibrous hydrogel for soft tissue engineering is demonstrated using gelatin–hydroxyphenylpropionic acid (Gel–HPA) by electrospinning and enzymatic crosslinking. Gel–HPA fibrous hydrogel was prepared by crosslinking the electrospun fibers in ethanol-water solution with an optimized concentration of horseradish peroxidase (HRP) and H_2_O_2_. The prepared fibrous hydrogel held the soft and elastic mechanical property of hydrogels and the three-dimensional (3D) fibrous structure of electrospun fibers. It was proven that the hydrogel scaffolds were biocompatible, improving the cellular adhesion, spreading, and proliferation. Moreover, the fibrous hydrogel showed rapid biodegradability and promoted angiogenesis in vivo. Overall, this study represents a novel biomimetic approach to generate Gel–HPA fibrous hydrogel scaffolds which have excellent potential in soft tissue regeneration applications.

## 1. Introduction

Each year, millions of patients worldwide suffer from the loss of soft tissue involving skin, fat, and muscle, because of trauma, tumor excision, congenital malformation, and aging [1]. However, the repair and regeneration of soft tissue is a ubiquitous clinical problem due to the clinical and material limitations [2]. Autologous tissue transfer, one of the most common surgical options, requires moving the same volume of tissue from another part of the body, resulting in donor site deficits [3], while prosthetic implants are prone to foreign body responses or immune rejection, leading to fibrosis and encapsulation [4]. Furthermore, even if the synthetic tissue scaffolds have been appropriately implanted, the functionality lost and the reduced graft survival remain challenges [5]. Because the extracellular matrix (ECM) plays a pivotal role in cell survival, migration and differentiation, numerous materials with ECM mimicking biophysical and biochemical properties have been developed to address these limitations [6,7,8,9].

Among the various kinds of tissue engineering scaffolds, hydrogels have been intensively studied for soft tissue engineering applications owing to their inherent priorities such as their native 3D structure and elastic properties, which are similar to natural soft tissues [10,11,12]. Rigid materials are not suitable for soft tissue regeneration and would cause a severe inflammatory response in vivo [13]. In addition, hydrogels with good injectability and proper viscoelastic properties have already been successfully used for central nervous regeneration and intervertebral disc repair after hybrid additive-manufactured poly (ε-caprolactone) scaffolds [14,15]. However, most of the reported hydrogel scaffolds were bulk hydrogels obtained by gelling single or multiple polymer solutions through physical or chemical reactions. The nanoscale network structure of these hydrogels may significantly affect cell activity and function, such as hindering cell spreading and migration [16,17].

Soft tissues can be viewed as a composite hydrogel matrix consisting of cells and reinforcing protein fibers [18]. Hence, many studies have been conducted to fabricate fibrous scaffolds to mimic the fibrous structure of native ECM [19,20]. The electrospinning technique remains one of the most convenient methods to produce fibrous scaffolds with architectural similarities to ECM. It provides versatility, allowing the use of different polymers to fabricate fibers with a controlled diameter, orientation, and a highly porous microstructure, through tuning the process parameters of electrospinning or in combination with other techniques [21,22,23]. Both synthetic and natural polymers have been used to fabricate electrospun nanofibers [24,25,26,27]. Among them, gelatin electrospun nanofibers have been attracting more and more attention due to their excellent biocompatibility, biodegradability and low immunogenicity [28]. However, gelatin electrospun membranes without crosslinking have weak mechanical properties and can be dissolved in water. Chemical crosslinking agents, such as glutaraldehyde and carbodiimide, have been used to stabilize gelatin fibers by crosslinking the abundant amino and carboxyl groups in the molecules [29,30]. Recently, gelatin methacryloyl (GelMA) electrospun nanofibers with elastic mechanical properties and adjustable degradation rate had been successfully designed for skin tissue regeneration [28,31]. This GelMA electrospun membrane could promote cell infiltration and facilitate vascularization by supporting the adhesion, proliferation and migration of endothelial cells and dermal fibroblasts. However, chemical crosslinkers and photoinitiators have shown potential cytotoxicity, thus limiting their biomedical applications [32,33,34].

Gelatin–hydroxyphenylpropionic acid (Gel–HPA) synthesized by incorporating HPA to the amine groups of gelatin molecules can be crosslinked through an enzymatic oxidation reaction. According to reports, enzymatic crosslinking has excellent biocompatibility without inducing cytotoxicity [35,36]. The HPA functionality maintains the superb biocompatibility of gelatin and additionally endows material stability and tunable mechanical properties after enzymatic crosslinking. For example, Gel–HPA bioactive bulk hydrogels with different stiffnesses have been manufactured and used for the 3D culture of chondrocytes and mesenchymal stem cells [37]. Moreover, Gel–HPA has also been produced into hollow hydrogel fibers by HRP crosslinking the precursor solution flowing within a capillary tube [38]. To further simulate the biophysical and microstructure of ECM, hydrogel electrospun fibers made of bioactive materials and crosslinked with low cytotoxic agents are highly required.

**Figure 1:**
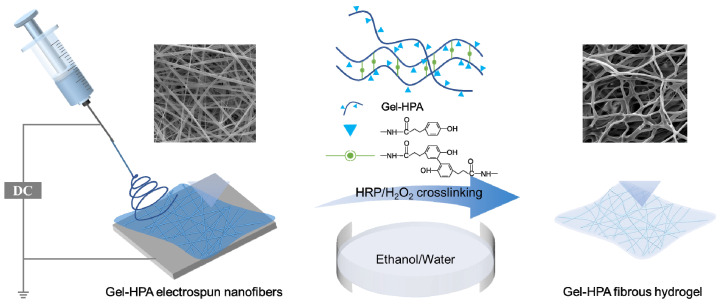
Preparation scheme of the gelatin–hydroxyphenylpropionic acid (Gel–HPA) fibrous hydrogel.

Herein, a new method for producing fibrous hydrogel scaffolds with dual properties of hydrogel softness and an electrospun fibrous structure through electrospinning and enzymatic crosslinking was reported. Furthermore, these fibrous hydrogels also possessed the characteristics of hydrogels with high water absorption and transparency (Figure 1). In this study, enzyme-crosslinkable Gel–HPA was synthesized and electrospun into nano-microfibers and then crosslinked in ethanol-water solution with HRP/H_2_O_2_. Then, the chemical component, morphology, water absorption and degradation of prepared Gel–HPA fibrous hydrogel were investigated. In order to explore the potential application in soft tissue engineering, the cell proliferation and viability on the scaffolds was assessed in vitro, and the biocompatibility and biodegradability of the scaffolds were also evaluated in vivo using a rat model.

## 2. Materials and Methods 

### 2.1. Synthesis of Gelatin–Hydroxyphenylpropionic Acid

Gelatin–hydroxyphenylpropionic acid (Gel–HPA) macromer was synthesized according to a previously described method [38]. Briefly, 1.32 g of hydroxyphenylpropionic acid (Sigma–Aldrich, USA) was dissolved in 40 mL dimethyl sulfoxide (DMSO, Aladdin Reagent, Shanghai, China), and then 60 mL of milli-Q water was added into the solution. Afterwards, 1.28 g of N-hydroxysuccinimide (NHS, Aladdin, Shanghai, China) and 1.52 g of 1-ethyl-3-(3-dimethylaminopropyl)- carbodiimide (EDC, Aladdin, Shanghai, China) were dissolved in the mixture, and the mixture was stirred at high rotation speed under the maintained pH of 4.5 at room temperature. After stirring for 3 h, 60 mL of 6.6% (*w*/*v*) gelatin solution was poured into the mixture and then stirred overnight at the pH of 4.5. The mixture was then extensively dialyzed against 100 mM of NaCl solution, 25% ethanol for 1 day each and Milli-Q water for 3 days using a dialysis membrane (12–14 kD molecular weight cut off, Spectrum Laboratories Inc., Rancho Dominguez, CA, USA). After dialysis, the Gel–HPA was lyophilized and stored at −20 °C for further use. 

### 2.2. ^1^H Nuclear Magnetic Resonance (NMR)

The synthesis of Gel–HPA macromers was confirmed using ^1^H NMR according to a previously described method [39]. ^1^H NMR spectra were collected using a Varian INOVA NMR spectrometer (Bruker, Billerica, MA, USA) with a single axis gradient inverse probe at a frequency of 300 MHz. Before the measurement, 20 mg of Gel–HPA dissolved into 1 mL of deuterium oxide containing 0.05% (*w*/*v*) 3-(trimethylsilyl)propionic-2,2,3,3-d4 acid sodium salt (Sigma-Aldrich, Saint Louis, MO, USA). The pristine gelatin without functionalization was also examined as a control. The experiment was independently repeated three times.

### 2.3. Electrospinning of Gel–HPA and Gelatin Electrospun Fibers

Electrospinning solution was prepared by dissolving 1.5 g of freeze-dried Gel–HPA sponge in 10 mL mixture of hexafluoroisopropanol (HFIP, Shanghai Macklin Biochemical Co. Ltd., Shanghai, China) and Milli-Q water (9:1, *v*/*v*) completely. The Gel–HPA solution was placed in the 10-mL syringe mounted on the microinjection pump. The voltage was set to 18 kV, a drum collector (10 cm diameter, 20 cm width, and 1000 n/min rotating speed) was placed 18 cm from the injector nozzle to collect random nano-microfibers, and the polymer solution was pumped out at a rate of 3 mL/h. The fiber membrane can be formed between the electrode rods after turning on the power, and an electrospun scaffold with a certain thickness can be obtained after consuming all the solutions. The electrospun fibers were stored for two days for the solvent evaporation. Gelatin electrospun nano-microfibers were also fabricated by using the same electrospinning process parameter as Gel–HPA fibers.

### 2.4. Crosslinking of Gel–HPA Fibrous Hydrogels and Gelatin Fibers

The Gel–HPA fibrous hydrogels were crosslinked by the enzymatic oxidative reaction of HPA moieties using horseradish peroxidase (HRP, Aladdin, Shanghai, China) and H_2_O_2_ (Aladdin, Shanghai, China), in which HRP was used as a catalyst for the oxidative coupling of phenol derivatives, proceeded at the C–C and C–O positions between phenols under mild reaction conditions. To prevent the dissolution of Gel–HPA or gelatin nano-microfibers before they were wholly crosslinked, the crosslinking reaction was conducted in ethanol-water solution (the ratio of ethanol to water were 95:5, 85:15, 75:25, *v*/*v*) where the HRP was 10 unit/mL and H_2_O_2_ was 100 mM. After a 24-h crosslinking reaction, the prepared scaffolds were repeatedly washed in phosphate-buffered saline (PBS) and then used for the following in vitro and in vivo experiments. 

Moreover, the influence of the concentration of H_2_O_2_ on the crosslinking reaction was investigated. The electrospun Gel–HPA fibrous hydrogels were crosslinked by the ethanol-water solution (85:15, *v*/*v*) which contained HRP (10 unit/mL) and various concentrations of H_2_O_2_ (10 mM, 50 mM, 100 mM). The other parameters and procedures were the same as before. The gelatin electrospun fibers were also crosslinked by 60 mM EDC and 12 mM NHS overnight in an alcohol-water solution with the same ratio as that used for the crosslinking of the Gel–HPA fiber. The experiment was independently repeated three times.

### 2.5. Scanning Electron Microscopy (SEM)

The morphologies of Gel–HPA and gelatin electrospun fibers before and after crosslinking were observed by SEM (FEI Quanta 250 FEG, Thermo Fisher Scientific, Waltham, MA, USA) at a 10-kV beam voltage. Gel–HPA and gelatin fibers before crosslinking were directly coated in platinum before imaging, and the crosslinked samples were freeze-dried before coating. The diameters of the nano-microfibers were obtained by using Image-J software based on the SEM images, and at least 100 fibers were analyzed.

### 2.6. Water Uptake Measurements

Freeze-dried Gel–HPA fibrous hydrogels and gelatin fibrous scaffolds were cut into a rectangle shape (1 cm × 2 cm) and weighted (W_0_). Then, the samples were immersed in PBS at 37 °C. After predetermined immersion times, the samples were blotted and immediately weighed to get the wet weight (W_t_). Gel–HPA scaffolds crosslinked in the solution of ethanol-water (85:15) containing HRP (10 unit/mL) and H_2_O_2_ (100 mM) were used for this experiment. The experiment was independently repeated three times. The water uptake (W) was calculated according to the equation:

(1) $$W=\frac{W_{t}-W_{0}}{W_{0}}\times 100\%$$

### 2.7. Mechanical Testing

Tensile testing was done on a universal tensile tester (UTM2230, Shenzhen SUNS Technology, Shenzhen, China) with the wet samples. To prevent shrinkage during crosslinking and crimp when fixing on the tester, specimens were cut into rectangular pieces (1 cm × 3 cm) and settled on frames before crosslinking and mechanical testing. The distance between clamps was around 2 cm after fixing and spreading the specimens. The scaffolds were tested at a strain rate of 3 mm/min. The Young’s moduli of the samples (*n* = 6) were calculated from the obtained stress/strain curves. The experiment was independently repeated three times.

### 2.8. Measurement of Enzymatic Degradation

Gel–HPA fibrous hydrogels and gelatin fibrous scaffolds (2 cm^2^ approximately) were prepared and weighed (W_0_). Then all the samples were immersed in 2 mL of PBS containing 0.1 mg/mL of collagenase type-I (Solarbio, Beijing, China) and incubated at 37 °C. After degradation for 0, 0.3, 0.6, 1, 1.5, 3, 4.5, 12, 24 and 36 h, the samples were washed using Milli-Q water and freeze-dried. After that, the samples were weighted (W_t_). The remaining weight after degradation was determined by normalizing the W_t_ to the initial dry weight W_0_. The experiment was independently repeated three times.

### 2.9. In Vitro Cell Viability, Spreading and Proliferation

Human umbilical vein endothelial cells (HUVECs) were incubated in an incubator with 5% CO_2_ at 37 ℃, and the Roswell Park Memorial Institute (RPMI) 1640 medium supplemented with 10% fetal bovine serum) was refreshed every day. After 90% confluence, a cell suspension with a complete medium was prepared using trypsin–ethylenediaminetetraacetic acid (EDTA) for dissociation for 3 min. Then, the cells were calculated and seeded with a density of 5000 cells/cm^2^ onto each sample. Before cell seeding, Gel–HPA fibrous hydrogels and gelatin fibrous scaffolds fixed with sterilized steel washers were soaked in a 75% ethanol solution for overnight, washed with PBS for three times and then exposed to UV light on each side for 30 min for sterilization.

Live/dead staining was performed using a Cellstain Double Staining Kit (NanJing KeyGen Biotech, Nanjing, China) to evaluate the cell viability in the scaffolds. After 1 day and 3 days of culture, the scaffolds containing cells were washed with PBS and incubated with serum-free medium containing calcein-acetoxymethyl ester (AM) (2 µM) and propidium iodide (4 µM) for 15 min. After 3 days of culture, the cell cytoskeleton was observed by F-actin staining. Briefly, the scaffolds containing cells were washed 3 times with PBS, fixed with 4% paraformaldehyde at 4 °C for 24 h, and washed twice with PBS. After that, scaffolds were then immersed in 5 mL of 0.2% Triton X-100 for 50 min to permeate the cells. After washing 3 times with PBS and blocking with 1% bovine serum albumin (BSA) solution for 30 min at room temperature, the sample was immersed in a 40-fold diluted fluorescent phalloidin stock solution (Biotium, Hayward, CA, USA) for 60 min to stain actin filaments. The cells were washed with Dulbecco’s phosphate-buffered saline (DPBS) three times and incubated in 4’,6-dimidyl-2-phenylindole dihydrochloride (DAPI) for 10 min at room temperature. The fluorescence images were captured using a confocal microscope (Zeiss LSM 510 Meta, Carl Zeiss Jena, Germany). The number of cells cultured after 1 and 3 days was determined using a cholecystokinin-8 (CCK-8) assay kit (Dojindo Laboratories, Tokyo, Japan) by measuring the absorbance at 450 nm. The experiment was independently repeated three times.

### 2.10. In Vivo Implantation

To evaluate the biocompatibility and in vivo degradation of the electrospun fibrous hydrogel, an animal experiment was carried out. Adult male Sprague Dawley (SD) rats (*n* = 3) were anesthetized with 3% sodium pentobarbital solution (30 mg kg^−1^). The crosslinked Gel–HPA fibrous hydrogels and gelatin fibrous scaffolds fixed on steel washers were sterilized and then subcutaneously implanted into the dorsal region of the rats under standard sterile operation. The steel washers without scaffolds were also placed in the subcutaneous tissue as a control group. After one month in vivo implantation, the tissues containing fibrous scaffolds were carefully separated from the mice’s tissues, washed with PBS three times and fixed with 10% neutral buffer formalin at room temperature for 2 days, dehydrated in a series of ethanol solutions with increasing ethanol concentrations from 70 to 99.5%, embedded in paraffin, then sectioned through the height of the scaffold. The cross-sections were deparaffinized and stained with hematoxylin and eosin (HE) and Masson stain.

All animal experiments were performed in compliance with the relevant laws and the Animal Experiment Committee guidelines of Zhengzhou University. All the animal procedures were approved by the Animal Experiment Committee of Zhengzhou University.

### 2.11. Statistical Analysis

All data are reported as the mean ± standard deviation (SD). Statistical analysis was performed using a one-way analysis of variance. All statistical analyses were executed using KyPlot 2.0 beta 15.

## 3. Results and Discussion

### 3.1. Synthesis of Gel–HPA

**Figure 2:**
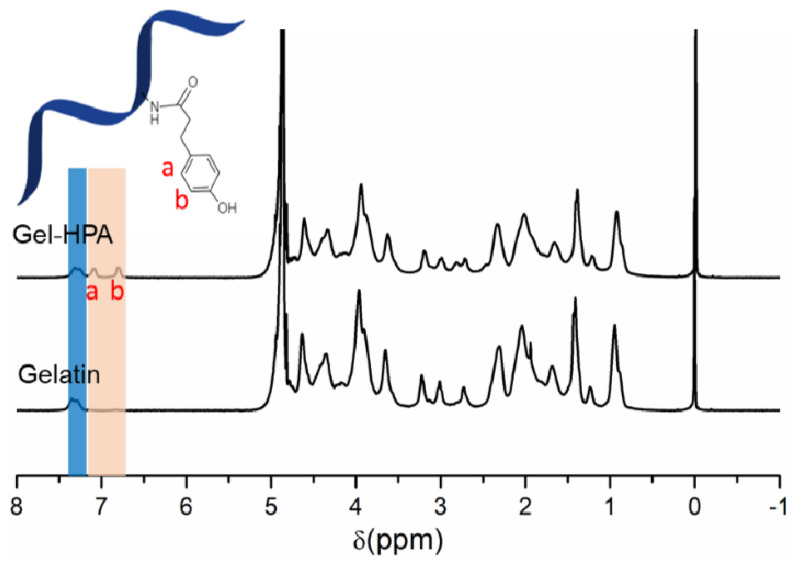
^1^H NMR spectra of pristine gelatin and Gel–HPA macromer.

Hydrogels composed of Gel–HPA macromers are attractive for tissue engineering and other biomedicine applications due to their high biocompatibility and controllable biophysical properties [40,41]. The Gel–HPA macromer was obtained by a general carbodiimide/active ester-mediated coupling reaction. The amine group in gelatin was conjugated with succinimide-activated HPA under an acidic environment. The successful functionalization of Gel–HPA was confirmed by using ^1^H NMR measurement. From the ^1^H NMR spectrum of Gel–HPA, the peaks at chemical shift (δ) 6.8 ppm and 7.1 ppm indicate the presence of the aromatic protons of HPA, in addition to the aromatic protons of phenylalanine and tyrosine residues of gelatin (7.3 ppm) (Figure 2).

### 3.2. The Influence of Crosslinking Parameters on Morphology of Electrospun Scaffolds

In this study, a Gel–HPA fibrous hydrogel scaffold was developed through electrospinning and horseradish peroxidase (HRP) crosslinking. The Gel–HPA macromer could be crosslinked through the oxidative coupling of the HPA moiety, which was catalyzed by hydrogen peroxide (H_2_O_2_) and HRP. The ethanol-water mixture was used as the crosslinking solvent because Gel–HPA and gelatin electrospun fibers without crosslinking will be dissolved in pure water immediately.

**Figure 3:**
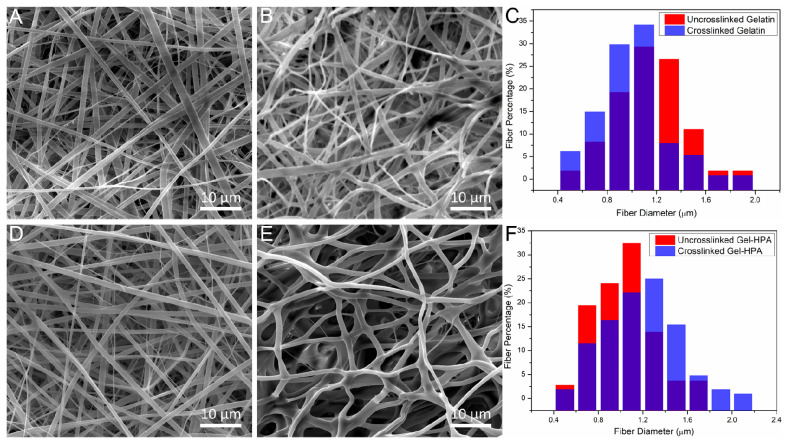
The morphologies of crosslinked gelatin fibrous scaffolds before (**A**) and after (**B**) crosslinking (ethanol-water, 85:15); the morphologies of Gel–HPA fibrous hydrogels before (**D**) and after (**E**) horseradish peroxidase (HRP) crosslinking (ethanol-water, 85:15, 10 unit/mL HRP and 100 mM H_2_O_2_). Fiber diameter changes of gelatin (**C**) and Gel–HPA (**F**) before and after crosslinking.

The microscopic morphology of gelatin and Gel–HPA scaffolds were examined by SEM. Furthermore, the preparation parameters for Gel–HPA fibrous hydrogel were optimized based on these results. As shown in Figure 3, the electrospun gelatin fibers and Gel–HPA fibers were straight and aligned randomly with similar fiber diameters before crosslinking. The crosslinked Gel–HPA fibers were curvy and sparsely arranged, which was different from Gel–HPA fibers before crosslinking (Figure 3D). The shape of the fibers changed from a strip to a cylindrical shape, and their diameter slightly increased, which may be caused by the crosslinking process and swelling when immersed in water, respectively, whereas the gelatin fibers maintained the shape of the strip, and there was no apparent change in the fiber diameter (Figure 3C). Moreover, gelatin exhibited a high packing density before and after crosslinking, which would render the cell infiltration. It is worth noting that the HRP crosslinked Gel–HPA fibers presented a highly porous matrix architecture, and the shape of fibers changed to rod from the strip (Figure 3E), while the crosslinked gelatin fibers were still tightly packed. The morphology of Gel–HPA may be caused by the HRP crosslinking strategy. Enzymatically crosslinked collagen mats were also reported to have a higher porosity than other chemical crosslinking methods, which was consistent with our study [42]. This porous structure is beneficial for cell seeding and ingrowth. All results indicated that the enzymatically crosslinked Gel–HPA scaffolds had a better microscopic morphology and porous architecture than the gelatin scaffolds.

**Figure 4:**
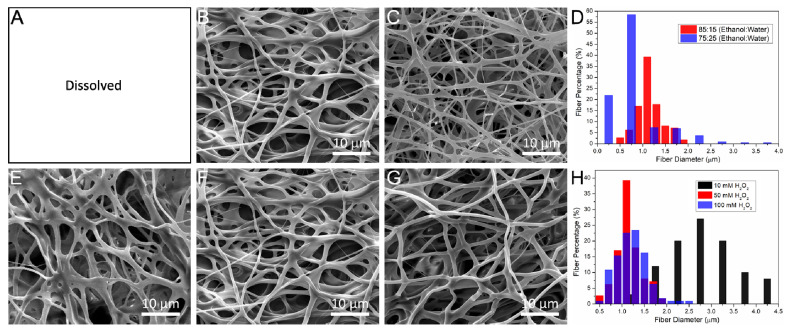
SEM images of Gel–HPA fibrous hydrogels prepared in different conditions: 10 unit/mL HRP, 50 mM H_2_O_2_ and different ratios of ethanol:water 95:5 (**A**), 85:15 (**B**) and 75:25 (**C**); 10 unit/mL HRP, ethanol–water solution (85:15), and different concentration of H_2_O_2_: 10 mM (**E**), 50 mM (**F**) and 100 mM (**G**); the influence of crosslinking solution (**D**) and H_2_O_2_ concentration (**H**) on fiber diameters.

The crosslinking conditions affect the microstructure of the Gel–HPA fibrous hydrogel and even determine whether the crosslinking can be successful. Therefore, the microstructure of Gel–HPA fibrous hydrogel under different crosslinking parameters was investigated based on the SEM results. With 10 unit/mL HRP and 50 mM H_2_O_2_, the proportion of ethanol and distilled water (95:5, 85:15, 75:25) was studied to optimize the crosslinking solvent based on the microscopic morphology. As shown in Figure 4, there were only SEM results of Gel–HPA scaffolds crosslinked in the ethanol-water solution (85:15, 75:25) because the sample crosslinked in 95:5 would dissolve once immersed in water, indicating unsuccessful crosslinking. It was believed that the reason was that high concentrations of ethanol might restrain the enzymatic activity and further inhibit the catalytic reaction. Though a porous architecture was presented in the samples crosslinked under the ratios of 85:15 and 75:25, the fiber diameter of the 85:15 group was larger than that of the 75:25 group (Figure 4D), which indicated that the Gel–HPA 85:15 fibers might be more robust and had a better water uptake property compared to the Gel–HPA 75:25 fibers. Therefore, the 85:15 proportion of ethanol-water solvent was chosen to optimize the crosslinking of Gel–HPA fibers.

Furthermore, under 85:15 of ethanol and distilled water, the H_2_O_2_ concentration (10 mM, 50 mM, 100 mM) was changed to explore its influence on the enzymatic crosslinking. As shown in Figure 4, the Gel–HPA (10 mM) and Gel–HPA (50 mM) fibers adhered to each other, exhibiting a high packing density and small pores. Therefore, the low concentration of H_2_O_2_ was not enough for the catalytic reaction, inducing the dissolution of Gel–HPA before valid crosslinking. The fibers of Gel–HPA crosslinked using 100 mM H_2_O_2_ arranged more loosely, and the pore size was also larger than other groups. This structure could facilitate cell migration along with the depth of the scaffolds and nutrient diffusion. Since there is almost no cytotoxicity of HRP, a relatively high concentration was used directly in this study without further optimization to ensure the complete reaction. Therefore, the Gel–HPA electrospun fibers crosslinked under ethanol-water mixture (the volume ratio of 85:15) with 100 mM H_2_O_2_ and 10 unit/mL HRP were chosen for the subsequent studies. 

### 3.3. The Physical Properties of Electrospun Scaffolds

**Figure 5:**
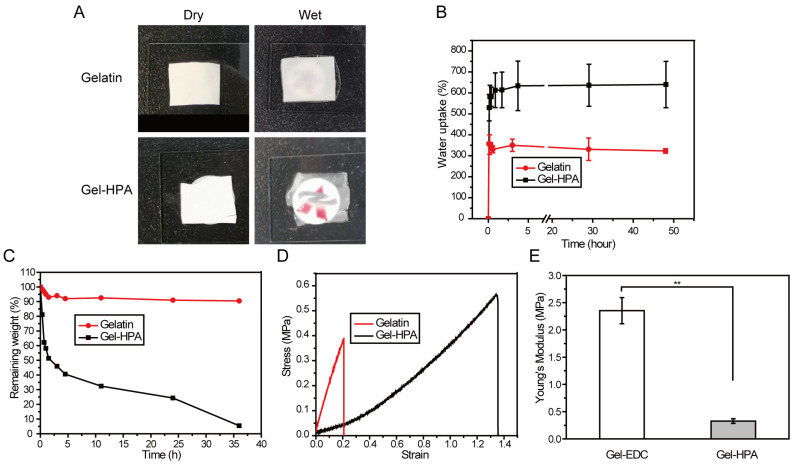
The gross appearance in a dry and wet state (**A**), water uptake (**B**), and enzymatic degradation (0.1mg/mL of collagenase) (**C**) of crosslinked gelatin fibrous scaffolds and Gel–HPA fibrous hydrogels. Stress and strain curve of tensile testing (**D**) and Young’s modulus (**E**) of crosslinked gelatin fibrous scaffolds and Gel–HPA fibrous hydrogels in the wet state. Data are presented as the means ± SD of three independent experiments. ** *p* < 0.01.

From the images in Figure 5A, it can be observed that Gel–HPA fibrous hydrogel was transparent, while the EDC/NHS crosslinked gelatin fibrous membrane kept its white color after immersing in water. Gel–HPA after crosslinking by HRP exhibited a gel-like state, which was possible due to its highly hydrophilic characteristics and the high water content. The water uptake of Gel–HPA fibrous hydrogels and gelatin scaffolds was measured to evaluate the water sorption capacity. From Figure 5B, it was observed that the water uptake increased quickly during the first few minutes for both groups due to the very high surface area of electrospun nano-microfibers. The water uptake of gelatin reached equilibrium in 10 min. In contrast, the swelling ratio of Gel–HPA increased gradually after the quick absorption period, reaching a plateau until it was soaked in PBS for 4 h. The equilibrium water of Gel–HPA (more than six times its original weight) was much higher than gelatin group (more than three times its dry weight), reaching a similar swelling ratio to the previous reported GelMA fibrous hydrogel [43], which indicates the hydrogel properties of Gel–HPA electrospun fibers. Since natural ECM exhibits a high water content, promoting the transportation of nutrients and metabolites, the Gel–HPA fibrous hydrogel may exhibit a better cytocompatibility. It is meaningful to investigate the biodegradability of tissue engineering scaffolds because degradation can provide space for cell proliferation and neotissue ingrowth [44,45]. Herein, collagenase I was used to accelerate the degradation of gelatin-based scaffolds. The remaining weights of Gel–HPA fibrous hydrogel and EDC/NHS crosslinked gelatin fibrous scaffolds with collagenase treatment were shown in Figure 5C. Gel–HPA fibrous hydrogel degraded more quickly than the gelatin group, which was beneficial for soft tissue regeneration.

For soft tissue engineering, such as fat and skin tissue regeneration, the scaffolds would be affected by the exogenous mechanical activities, as well as the squeezing and pulling effect of the surrounding tissues. Therefore, the elasticity and stretch of the scaffolds are pivotal for the maintenance of long-term structural stability. Both Gel–HPA hydrogel and gelatin scaffolds exhibit the typical stress-strain curve of viscoelastic materials, as indicated by the tensile test. Gel–HPA fibrous scaffolds exhibited higher stress and elongation at break, indicating that Gel–HPA fibrous hydrogel was more elastic compared with the gelatin group. Its maximum tensile length went up to 1.35 times that of the original, which was more than six times larger than that of the gelatin scaffold (0.21 times) (Figure 5D). Additionally, the Young’s modulus of the Gel–HPA fibrous hydrogel was significantly lower than that of the gelatin fibrous scaffold (*p* < 0.05) (Figure 5E), which indicated that the Gel–HPA fibrous hydrogel had softer and more elastic mechanical properties, meeting the requirement for soft tissue engineering [46]. Moreover, it was reported that a soft matrix with a quick degradation rate could provide more space for cell proliferation and new tissue deposition, promote angiogenesis and improve blood vessel formation, which is vital for soft tissue regeneration [47,48].

### 3.4. Cell Viability, Spreading and Proliferation on Electrospun Scaffolds

**Figure 6:**
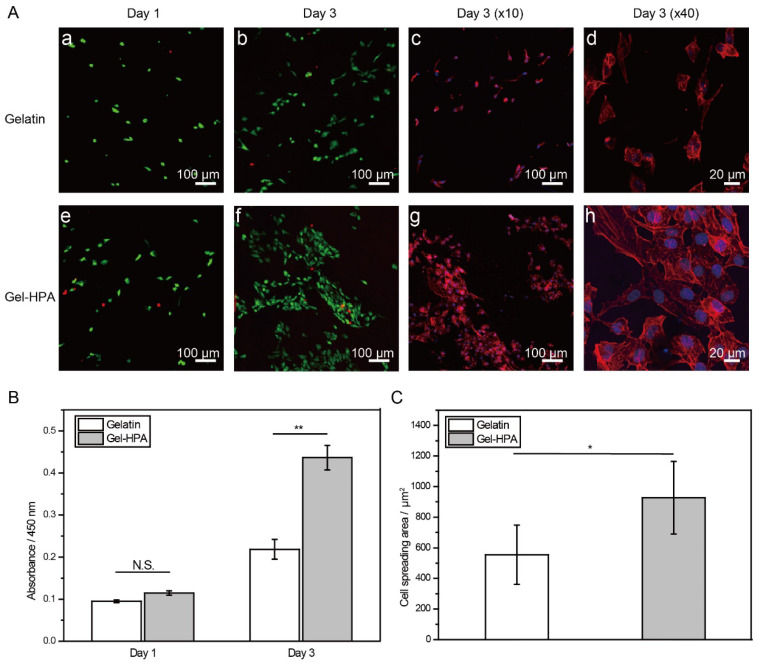
Live/dead staining (**a**,**b**,**e**,**f**. Green: live cells; red: dead cells) and F-actin staining (**c**,**d**,**g**,**h** Red: F-actin; blue: cell nuclei) of cells cultured in gelatin fibrous scaffolds and Gel–HPA fibrous hydrogels for 1 day and 3 days (**A**). Cell proliferation, *n* = 3 (**B**) and cell spreading area, *n* = 30 (**C**) on gelatin fibrous scaffolds and Gel–HPA fibrous hydrogels. Data are presented as the means ± SD of three independent experiments. * *p* < 0.05; ** *p* < 0.01.

The biocompatibility of this fibrous hydrogel was evaluated by live/dead cell staining and a CCK-8 cell proliferation assay. HUVECs were chosen due to them participating in angiogenesis, vascular homeostasis process, and soft tissue regeneration process. The gelatin electrospun nano-microfibers crosslinked by using EDC/NHS were used as controls. High cell viability in Gel–HPA and gelatin was observed, indicating the good biocompatibility of these two kinds of materials and their crosslinking approaches (Figure 6A). A higher cell density was observed after 3 days of incubation. This result was confirmed by the CCK-8 cell proliferation assay. The metabolic rate of cells on the Gel–HPA scaffold was significantly higher than that of cells on the gelatin group (*p* < 0.05) on day 3 (Figure 6B). This enhanced cell proliferation was mainly due to the high porosity and the low stiffness of Gel–HPA fibrous hydrogel. Many reports indicated that Gel–HPA derivatives are biocompatible, and their degradation products are small molecules and are relatively non-toxic [49]. Though the high concentration of H_2_O_2_ during the crosslinking of Gel–HPA was toxic, it can be removed by repeated washing before cell seeding. Overall, both the biocompatibility and cellular suitability of Gel–HPA fibrous hydrogel were better than the gelatin fibrous scaffold in vitro.

Moreover, F-actin fluorescence staining confirmed that the cells cultured in Gel–HPA fibrous hydrogel scaffold formed more actin filaments, and the cell spreading was enhanced, as indicated by the larger cell-spreading area than the one observed for cells cultured in gelatin fibrous scaffolds (Figure 6A,C). This phenomenon agreed with a previous study [50] in which mesenchymal stem cells cultured on HRP crosslinked hydrogel substrates developed longer focal adhesions and larger spreading areas than the cells cultured on hydrogel crosslinked with visible light. In that study, the enhanced cell spreading on enzyme-crosslinked materials was proven after excluding the other biophysical and biological factors by comparing the cell morphology on the substrates with a similar stiffness and arginine–glycine–aspartate (RGD) density. The strong actin filaments and high cell spreading area usually represent phenotypes for angiogenesis and vascularization [51,52]. Overall, though both Gel–HPA fibrous hydrogel and gelatin fibrous scaffolds showed excellent biocompatibility, Gel–HPA fibrous hydrogel crosslinked by HRP had the ability to enhance cell adhesion, cell spreading and cell proliferation, which are necessary for cell activities and tissue regeneration.

### 3.5. Histological Staining

**Figure 7:**
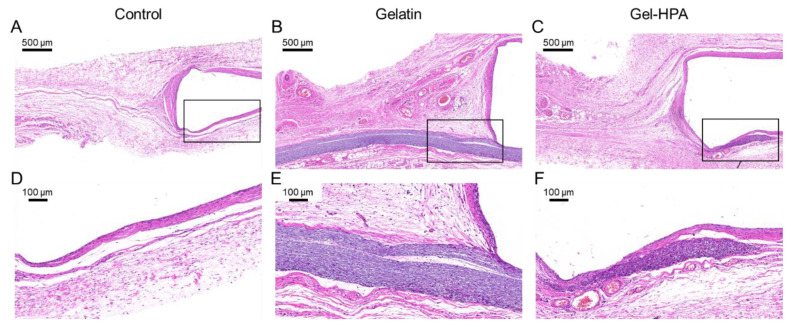
Hematoxylin and eosin (HE) staining of control group (**A**,**D**), gelatin fibrous scaffolds (**B**,**E**) and Gel–HPA fibrous hydrogels (**C**,**F**) implanted in vivo for 1 month.

Considering the requirements of biological and biodegradable properties for soft tissue engineering, in vivo implantation was conducted to investigate the performance of Gel–HPA fibrous hydrogel scaffolds. Specifically, Gel–HPA fibrous hydrogels and gelatin fibrous scaffolds were fixed on a steel washer before subcutaneous implantation. A steel washer without a scaffold was also implanted as a blank control. After 4 weeks of in vivo culture, the histological analysis was done by harvesting all the samples for HE staining. According to the results, it was found that Gel–HPA fibrous hydrogel was almost completely degraded after 4 weeks of implantation, and new tissues started replacing the scaffolds (Figure 7). Few areas of the Gel–HPA scaffolds near the steel washer had not been degraded.

Moreover, it can be observed that many cells penetrated inside the residue Gel–HPA fibrous hydrogel, with no apparent inflammation being detected. In contrast, the gelatin scaffold still maintained its original shape and size, and the cell penetration depth was limited, indicating the prolonged degradation time even in vivo and the high pack density of the gelatin fibrous scaffold. This result showed that Gel–HPA fibrous hydrogel had a more natural ECM-like structure and provided a suitable degradation rate for tissue regeneration compared with gelatin fibrous scaffolds. The cells surrounding Gel–HPA scaffolds penetrated inside the construct and synthesized new tissues during the degradation.

**Figure 8:**
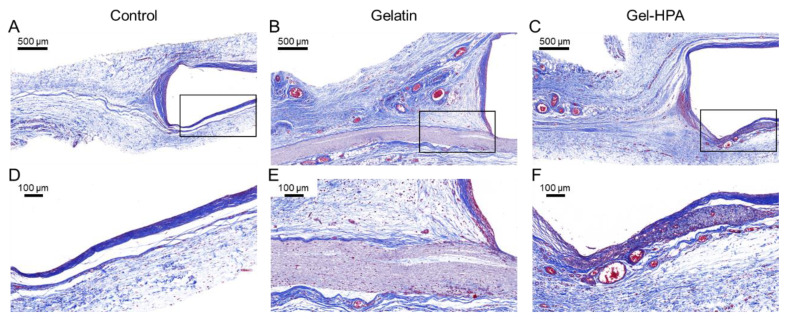
Masson staining of control group (**A**,**D**), gelatin fibrous scaffolds (**B**,**E**) and Gel–HPA fibrous hydrogels (**C**,**F**) implanted in vivo for 1 month.

The collagen capsules of the tissue gradually form at the interface between tissue and implant materials due to the inflammatory response. Masson trichrome stain, which stains collagen blue, was used to evaluate capsule formation after subcutaneous implantation for 1 month (Figure 8) [53]. As shown in Figure 8B,C, crosslinked electrospun gelatin scaffold was still nondegraded, whereas the Gel–HPA fibrous hydrogel was almost entirely biodegraded after in vivo implantation for one month. Furthermore, there was organized and bundled collagen around the gelatin scaffold, while there were tiny collagen fibers in Gel–HPA groups. Specifically, the collagen fibers disappeared in the degraded area, leaving the regenerated tissues, like in the blank control group. This result indicated that Gel–HPA nanofibrous hydrogel with soft mechanical properties and rapid degradation did not cause extensive inflammation. Interestingly, it was observed that there were many vascular structures near the Gel–HPA scaffold and inside the residue Gel–HPA scaffolds, which was potentially beneficial for soft tissue regeneration (Figure 8C). The microvascular structure inside the Gel–HPA construct could be observed clearly in the magnified image (Figure 8F). However, there was no vascular structure inside the gelatin fibrous scaffold. Moreover, Gel–HPA can be degraded by protease, and its degradation rate was much faster than that of gelatin. Vascularization was accelerated by the Gel–HPA fibrous hydrogel because of its quick degradation and soft elasticity, which was consistent with previous studies [54,55]. Sun et al. reported that softer dextran hydrogel could significantly stimulate stem cells to secrete angiogenic factors and promote vascularization [55].

Electrospun nanofibrous scaffolds are drawing widespread attention in tissue engineering due to their ability to mimic the fibrous structure of native ECM. However, most electrospun nanofibers are either non-degradable or degrade hydrolytically rather than degrading proteolytically like natural ECM [56]. Furthermore, the electrospinning of synthetic polymers often results in rigid and solid nanofibers, which hinder their application in soft tissue engineering. Gel–HPA, a derivative of gelatin, can be degraded by matrix metalloproteinases. In addition, our HRP enzymatic crosslinked gelatin fiber can swell in water and become transparent, showing hydrogel characteristics. Recently, a GelMA fibrous hydrogel scaffold crosslinked by photopolymerization was reported to have the ability to promote the regeneration of skin and the spinal cord rapidly [28,31]. The presented GelMA electrospun hydrogel fibrous scaffold also exhibits elastic mechanical properties and biodegradable properties. It can support endothelial cell adhesion, proliferation, and facilitate vascularization due to its fibrous structure and soft mechanical properties. Although the reported GelMA scaffold exhibits a similar swelling ratio and 3D fibrous structure to Gel–HPA, the elongation at break of randomly oriented GelMA fibers (around 60%) was significantly less than Gel–HPA fibrous hydrogel (135%) [31]. Moreover, the mild and bioactive HRP crosslinking process and the low cytotoxicity of residue chemicals make Gel–HPA fibrous hydrogel have more potential applications in soft tissue engineering.

## 4. Conclusions

Gel–HPA fibrous hydrogel was prepared for the first time through electrospinning and the HRP crosslinking reaction. The fibrous hydrogel with an ECM-like fibrous structure and high water content showed excellent biocompatibility because of the natural polymer gelatin and the low cytotoxicity of the enzymatic crosslinking strategy. It was fascinating that the electrospun fibers became transparent after HRP enzymatic crosslinking in the ethanol-water mixture. Meanwhile, the Gel–HPA fibrous hydrogel scaffolds also possessed high stretch and elasticity, which are beneficial for soft tissue regeneration applications. In addition, the fibrous hydrogel can promote endothelial cell adhesion and spread due to the crosslinking strategy. Compared with the EDC/NHS crosslinked gelatin fibers, the Gel–HPA fibrous hydrogel exhibited quick biodegradation and could be utterly biodegraded after 4 weeks of in vivo implantation. Most importantly, the Gel–HPA scaffold did not show visible adverse effects and had the function of promoting vascularization, which is vital for soft tissue regeneration. All in all, the Gel–HPA electrospun fibrous hydrogels developed in our study have many advantages for soft tissue engineering applications.
